# High mechanical strain of primary intervertebral disc cells promotes secretion of inflammatory factors associated with disc degeneration and pain

**DOI:** 10.1186/ar4449

**Published:** 2014-01-23

**Authors:** Rahul Gawri, Derek H Rosenzweig, Emerson Krock, Jean A Ouellet, Laura S Stone, Thomas M Quinn, Lisbet Haglund

**Affiliations:** 1 The Orthopaedics Research Lab, Department of Surgery, McGill University, 1650 Cedar 663 Avenue, Montreal, QC H3G 1A4, Canada; 2 McGill Scoliosis & Spine Group, Department of Surgery, McGill University, 1650 Cedar 663 Avenue, Montreal, QC H3G 1A4, Canada; 3 Alan Edwards Centre for Research McGill University, 740 Dr. Penfield Avenue, Montreal, QC H3G 0G1, Canada; 4 Faculty of Dentistry, McGill University, 3640 University Street, Montreal, QC H3A 0C7, Canada; 5 Soft Tissue Biophysics Lab, Department of Chemical Engineering, McGill University, 3610 University Street, Montreal, QC H3A 2B2, Canada; 6 McGill University Health Centre, Department of Surgery, Montreal General Hospital, Room C9.173, 1650 Cedar Avenue, Montreal, QC H3G 1A4, Canada

## Abstract

**Introduction:**

Excessive mechanical loading of intervertebral discs (IVDs) is thought to alter matrix properties and influence disc cell metabolism, contributing to degenerative disc disease and development of discogenic pain. However, little is known about how mechanical strain induces these changes. This study investigated the cellular and molecular changes as well as which inflammatory receptors and cytokines were upregulated in human intervertebral disc cells exposed to high mechanical strain (HMS) at low frequency. The impact of these metabolic changes on neuronal differentiation was also explored to determine a role in the development of disc degeneration and discogenic pain.

**Methods:**

Isolated human annulus fibrosus (AF) and nucleus pulposus (NP) cells were exposed to HMS (20% cyclical stretch at 0.001 Hz) on high-extension silicone rubber dishes coupled to a mechanical stretching apparatus and compared to static control cultures. Gene expression of Toll-like receptors (TLRs), neuronal growth factor (NGF) and tumour necrosis factor α (TNFα) was assessed. Collected conditioned media were analysed for cytokine content and applied to rat pheocromocytoma PC12 cells for neuronal differentiation assessment.

**Results:**

HMS caused upregulation of TLR2, TLR4, NGF and TNFα gene expression in IVD cells. Medium from HMS cultures contained elevated levels of growth-related oncogene, interleukin 6 (IL-6), IL-8, IL-15, monocyte chemoattractant protein 1 (MCP-1), MCP-3, monokine induced by γ interferon, transforming growth factor β1, TNFα and NGF. Exposure of PC12 cells to HMS-conditioned media resulted in both increased neurite sprouting and cell death.

**Conclusions:**

HMS culture of IVD cells *in vitro* drives cytokine and inflammatory responses associated with degenerative disc disease and low-back pain. This study provides evidence for a direct link between cellular strain, secretory factors, neoinnervation and potential degeneration and discogenic pain *in vivo*.

## Introduction

Treatment of chronic illnesses such as osteoarthritis (OA) and low-back pain presently consumes a significant amount of healthcare budgets worldwide. The current healthcare focus is shifting towards a better understanding of the underlying causes and pathological mechanisms of such diseases in order to generate better treatment strategies, decrease morbidity and reduce the overall economic impact. Low-back pain is a debilitating disorder requiring a significant amount of resources for treatment and is currently ranked sixth amongst the costliest treatable chronic illnesses [1]. Low-back pain is often attributed to intervertebral disc (IVD) degeneration, synonymously referred to as *degenerative disc disease* (DDD). Commonly implicated factors associated with degeneration include upregulated inflammatory cytokines, loss of resident cell populations, altered biomechanics, uneven loading of the spine and excessive mechanical stretch of tissue and cells [2]. Disc degeneration is a complex process, and the underlying mechanical and molecular mechanisms remain poorly understood.

The human lumbar spine is a partially flexible structure with six degrees of freedom in motion that is capable of a wide variety of routine and athletic movements [3]. Under various circumstances, the lumbar spine can be exposed to excessive or high mechanical strain (HMS) in the IVDs, resulting in tremendous local shear or compressive forces that cause tears in rings of the annulus fibrosus (AF). Severe cases of adverse strain can result in annular ruptures, nuclear herniation and nuclear extrusions [4]. Excessive mechanical strains can create a local proinflammatory microenvironment with a relatively concentrated release of cytokines [5,6]. The released cytokines in turn lead to protease production and activation, which initiate disc matrix degradation [7]. Thus a cascade of events is triggered, leading to disc degeneration characterised by loss of disc height, neuronal compression and radicular pain. In addition to increased cytokine expression, disc degeneration is associated with increased innervation in the normally aneural disc [8,9]. Evaluation of the interplay of mechanical strain to both disc tissue and disc cells and its effect on proinflammatory metabolite production is therefore important to fully understand the mechanisms of disc degeneration and to formulate recommendations for inhibiting disease progression.

Toll-like receptors (TLRs) are part of a superfamily of interleukin 1 (IL-1) receptors. They mainly serve to detect outer pathogen components such as peptidoglycan from Gram-positive bacteria and lipopolysaccharide from Gram-negative bacteria [10], as well as cartilage matrix fragments [11], thus triggering the innate immune response. Cytokine secretion is often a by-product of TLR activation, contributing to inflammatory responses [12]. Additionally, mechanical strain can promote increased TLR expression in chondrocytes and lung fibroblasts [13,14]. The increased mechanical strain in cartilage (as observed in OA) may therefore increase matrix fragments that promote TLR signalling, cytokine release and inflammatory responses. We have observed TLR2 and TLR4 expression in IVDs; however, the role of adverse mechanical strain on TLR expression and inflammatory cytokine production in IVDs has not been established.

We have previously developed a novel culture technique that facilitates more continuous growth of cells while limiting effects of contact inhibition and reducing the necessity for passaging [15-17]. High-extension silicone rubber (HESR) dishes are chemically modified to promote cell adhesion, then coupled to an iris-like device that uniformly expands the culture surface by up to 1,000%. With this culture device, cells are grown on a continuously expanding culture surface area as the cell population grows. Additionally, we have shown that low-frequency, high-magnitude cyclical strain applied by this device can modulate stem cell differentiation and lineage specification [18,19]. Because the culture surface can be expanded to magnitudes approaching injurious strain [20,21]. We hypothesized that high-amplitude cyclical mechanical strain (20% at a low frequency of 0.001 Hz) of AF and nucleus pulposus (NP) cells can directly promote inflammatory factor secretion associated with spinal disc degeneration, innervation and pain.

## Methods

### Source of tissue

This study was approved by McGill University’s Institutional Review Board (IRB A04-M53-08B) for a project titled “Human Intervertebral Discs used for Culture and Extracellular Matrix”. Family consent for tissue use was obtained in collaboration with the provincially run Transplant Quebec organ donation program. Three human lumbar spines were harvested in total. A total of five discs each from human donors were obtained (age range 19 to 22 years, mean age 20.5 years). Strict selection criteria were followed to ensure that only healthy IVDs were selected for this study. Donors with a history of back pain, spinal deformities or any cancer or radiation therapy were excluded. All the spines were scanned by X-ray, and the IVDs were visually inspected for signs of degeneration prior to cell isolation. The harvest procedure was performed within 6 hours of clamping the aorta, and cells were isolated within 8 hours.

### Human IVD cell isolation and culture

Human IVDs were separated from the adjoining vertebral body with a scalpel and divided into NP and the inner AF. Cells were enzymatically isolated from the separated regions as previously described [22]. NP and AF tissues were dissected into approximately 2-mm-thick pieces, washed twice in phosphate-buffered saline (PBS) containing 50 μg/ml gentamicin, 100 μg/ml penicillin, 100 U of streptomycin and 0.25 μg/ml amphotericin B. The tissues were digested with 0.2% PRONASE Protease (Calbiochem, Darmstadt, Germany) for 1 hour, then with collagenase type IA at 0.01% for NP and 0.04% collagenase type II for AF tissue for 4 hours in serum-free Dulbecco’s modified Eagle’s medium (DMEM). Passage 0 (P0) cells were used for the experiments. Cells were cultured at 37°C in 5% CO_2_ in DMEM containing 4.5 g/L glucose and supplemented with 10% foetal calf serum, 25 mmol/L 2-[4-(2-hydroxyethyl)piperazin-1-yl]ethanesulfonic acid, 0.25 μg/ml amphotericin B, 50 μg/ml L-ascorbate, 2 mmol/L GlutaMAX medium (Gibco/Life Technologies, Burlington, ON, Canada) and 50 μg/ml of gentamicin sulphate.

### Quantification of cell viability, cell proliferation and apoptosis

Glass coverslips were coated with about 100 μl of silicone (Factor II, Lakeside, AZ, USA) and cured for 2 hours at 70°C as described previously [16]. Briefly, the surface was rinsed with 30% sulphuric acid for 15 minutes and washed copiously with deionized water, followed by salinization with 1% (3-aminopropyl)triethoxysilane for 2 hours at 70°C. After another wash with water, the surface was functionalized with 6% (wt/wt) glutaraldehyde, then coated with 2 ml of monomeric rat tail collagen type I (50 mg/ml). Noncoated coverslips were also treated with 2 ml of monomeric collagen type I to serve as a control. IVD cells (10,000/cm^2^) were seeded and cultured in DMEM for 48 hours, then fixed in 4% paraformaldehyde for 10 minutes at room temperature. The cells were permeabilized and blocked (PBS, 0.1% Triton X-100, 1% bovine serum albumin) for 30 minutes and probed with antibodies against phosphohistone H3 (5 to 10 μg/μl, 1:250 dilution; Sigma-Aldrich, St Louis, MO, USA) or cleaved caspase 3 (3 to 6 μg/μl, 1:250 dilution; Sigma-Aldrich). Alexa Fluor 568 goat anti-rabbit immunoglobulin G (IgG) (3.5 to 6.5 μg/μl, 1:250 dilution; Sigma-Aldrich) was used as the secondary antibody. Samples were washed and mounted with Fluoroshield and 4′, 6-diamidino-2-phenylindole histology mounting medium (Sigma-Aldrich) and visualised on an Olympus IX81 motorized inverted fluorescence microscope (Olympus America, Center Valley, PA, USA). All images were captured using a 10× lens objective with MAG Biosystems software 7.5 (Photometrics, Tucson, AZ, USA). Three random positions per slide were obtained from three independent experiments. Positively stained nuclei were counted and plotted as the percentage of total nuclei. For viability assays, a separate set of live cultured cells from the same individual donors were cultured on the indicated surfaces (untreated glass coverslips or glass coverslips coated with functionalized silicone for 48 hours at the same time as samples used in the cellular apoptosis and proliferation assessments described above). Cell viability was assessed by Live/Dead Cell Viability Assay (Invitrogen, Carlsbad, CA, USA) according to the vendor’s instructions, and total dead and live cells were counted from three random positions in three independent experiments using images obtained with the fluorescence microscope described above.

### Static silicone and high-magnitude strain

IVD cells from NP and AF regions were seeded at a density of 250,000 cells/dish on HESR culture dishes (28-cm^2^ initial surface area) and static 60-mm polystyrene culture dishes coated with approximately 500 μm of silicone rubber termed *static silicone* (SS) (28-cm^2^ surface area; Factor II). The SS and HMS dishes were cultured for 48 hours with cells in their respective media prior to starting stretch protocols, as described above, supplemented with 10% foetal bovine serum (FBS). All silicone surfaces were chemically modified to promote cell adhesion as previously described [16,19].

After IVD cells were seeded for 48 hours, the growing surface was rinsed twice with sterile PBS and the respective culture media were replaced with serum-free medium supplemented with 1 ml/L of ITS (Insulin-Transferrin-Selenium; Gibco/Life Technologies). The resulting conditioned media were collected at the end of each experiment and stored in aliquots at −80°C for use in cytokine arrays and for PC12 cell analysis. With SS culture (28 cm^2^-surface area) used as a control, HMS cultures were subjected to 20% dynamic strain for 8 hours at a frequency of 0.001 Hz, followed by 16 hours of intervening rest for 48 hours. This slow strain rate allowed cells to be constantly strained without induction of apoptosis and cell death. Two protocols for mechanical stretching were followed for human NP and AF cells: 8 hours/16 hours/8 hours and 8 hours/16 hours/8 hours/16 hours. The experiments were ended at various time points, depending upon the protocol, then media were collected, cells were lysed and the lysate was collected for quantitative PCR (qPCR) analysis.

### RNA isolation

Cells from the above surfaces were lysed using 1 ml of lysis buffer (RNeasy Mini Kit; QIAGEN, Toronto, ON, Canada) for each surface and homogenized with a cell scraper, then lysate was collected in RNAse-free tubes. Total RNA was extracted using an RNeasy Mini Kit according to the manufacturer’s instructions. RNA concentrations and purity were determined by measuring A_260_ by calculating the A_260_/A_280_ ratio using a microplate reader (Infinite M200 PRO NanoQuant; Tecan, Männedorf, Switzerland).

### Real-time PCR

Total RNA isolated from the cells from each group was digested with DNase I and used for reverse transcription using the Omniscript Reverse Transcription Kit (QIAGEN). cDNA from 1 μg of RNA equivalent was mixed with random primers to a final volume of 20 μl and used in each reaction well during real-time PCRs using TaqMan chemistry run with the 7500 Fast Real-Time PCR System (Applied Biosystems, Foster City, CA, USA). Commercially available TaqMan array primers were used, and genes analysed were TLR2 (Hs006101101_m1), TLR4 (Hs01060206_m1), neuronal growth factor (NGF) (Hs00171458_m1) and tumour necrosis factor (TNF) (Hs00174128_m1), with 18S (Hs99999901_s1) used as the endogenous control. Gene expression in all samples was first normalized to 18S, then compared between stretched and static samples. Gene expression was calculated using the 2^−ΔΔCt^ method [23].

### Cytokine array analysis

Collected conditioned media from static and HMS cultures were incubated over commercially available human cytokine antibody arrays according to the manufacturer’s instructions (RayBiotech, Norcross, GA, USA). Chemiluminescence detection was performed using the reagent provided in the array kit and visualized with the ImageQuant LAS 4000 digital imaging system (GE Healthcare Life Sciences, Pittsburgh, PA, USA). ImageQuant TL software was used for pixel quantification. Background baseline noise was subtracted from all samples, and the results expressed are as relative ratios compared to controls.

### Induction of neurite outgrowth using conditioned media

For all experiments, 2 × 10^5^ PC12 cells/well (American Type Culture Collection, Manassas, VA, USA) were seeded onto six-well culture dishes coated with 50 μg/ml collagen type I and 0.1% poly-L-lysine (70 to 150 kDa; Sigma-Aldrich). Cells were allowed to attach to culture surfaces for 24 hours in RPMI 1640 medium supplemented with 1% antibiotic/antimycotic solution, 5% FBS and 10% horse serum (all obtained from Gibco/Invitrogen). After cell attachment, control wells were changed to 0.1% serum RPMI 1640 medium supplemented with 50 ng/ml recombinant βNGF (BioShop Canada, Burlington, ON, Canada) or sterile water vehicle (control). The remaining wells were subjected to 1.5 ml of conditioned media collected from the experiments described above (SS and HMS AF cultures and SS and HMS NP cultures). Neurite outgrowth was monitored for 4 days. Three random phase images per sample were taken from each individual experiment (*n* = 3), and the neurites per cell body were counted and scored as either ≤3 or >3 neurites/cell. Phase images were captured using a Zeiss Axiovert 40C microscope (Carl Zeiss Microscopy, Thornwood, NY, USA) equipped with a Canon PowerShot A640 digital camera (Canon USA, Melville, NY, USA) attached to a Zeiss MC 80 DX 1.0× tube adapter (Carl Zeiss Microscopy). LIVE/DEAD assays were performed and quantified as described above.

### Other materials

Mechanical stretching device and HESR dishes were bought from Cytomec GmbH (Spiez, Switzerland). PRONASE Protease was obtained from Calbiochem. Collagenase type IA, GlutaMAX, NaCl, Na-citrate, (3-aminopropyl)triethoxysilane, phosphohistone H3, cleaved caspase 3, goat anti-rabbit IgG, monomeric rat tail collagen type I and ethylenediaminetetraacetic acid were purchased from Sigma-Aldrich. Silicone rubber was purchased from Factor II (A-221-05 LSR). Penicillin/streptomycin, gentamicin sulphate, amphotericin B, DMEM, ITS and FBS were obtained from Gibco/Life Technologies. The RNeasy Mini Kit for RNA isolation and Omniscript Reverse Transcription Kit were purchased from QIAGEN (); lysis buffer, RT-PCR kit, TaqMan qPCR MasterMix and TaqMan primer probes were purchased from Applied Biosystems. Human Cytokine Array Q1 was obtained from RayBiotech (Burlington, ON, Canada).

### Statistical analysis

All values are represented as means ± SEM of at least three independent experiments from three different human donors for each experiment. Differences between experimental groups were assessed using a two-tailed Student’s *t*-test with the *post hoc* Bonferroni correction. Differences were considered significant at *P*< 0.05.

## Results

### IVD cell growth on modified silicone surfaces

**Figure 1:**
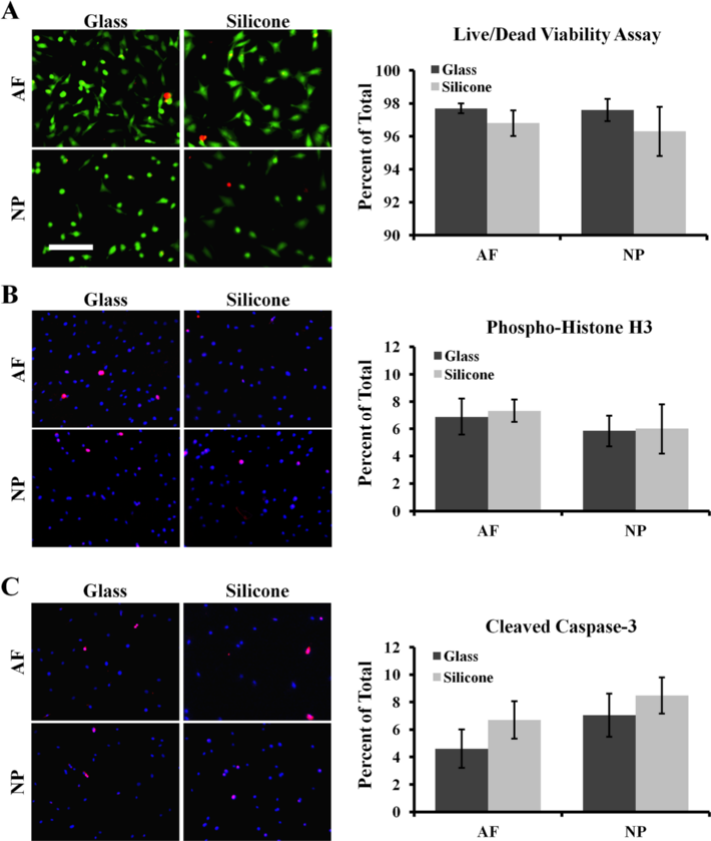
**Viability and proliferation of intervertebral disc cells cultured on modified silicone surfaces. (A)** Representative images show living (green) and dead (red) cells, with quantification shown at right. **(B)** Representative immunofluorescence images of phosphohistone H3 stained cells (red) and 4′,6-diamidino-2-phenylindole (DAPI) nuclear stain (blue), with quantification shown at right. **(C)** Representative immunofluorescence images of cleaved caspase 3–stained cells (red) and DAPI nuclear stain (blue), with quantification shown at right. AF, Annulus fibrosus; NP, Nucleus pulposus. Scale bar: 200 μm. Error bars represent SEM. Three independent experiments were performed. Significance was calculated by Student’s *t*-test.

Prior to examining the dynamic IVD cell culture, the effects of treated silicone surfaces on IVD cell viability, proliferation and apoptosis were determined. NP and AF cells were cultured on uncoated or silicone-coated glass coverslips. LIVE/DEAD assay and immunofluorescence microscopy were used to assess phosphohistone H3 and cleaved caspase 3 (Figure 1). No significant differences in NP or AF viability were observed between coated and uncoated surfaces. No difference in phosphohistone H3 activity was detected between coated and uncoated surfaces for NP or AF cells. A nonsignificant trend towards increases in cleaved caspase 3 activity was observed in both NP and AF cells in SS culture.

### High mechanical strain induces proinflammatory and pronociceptive gene expression in human IVD cells

**Figure 2:**
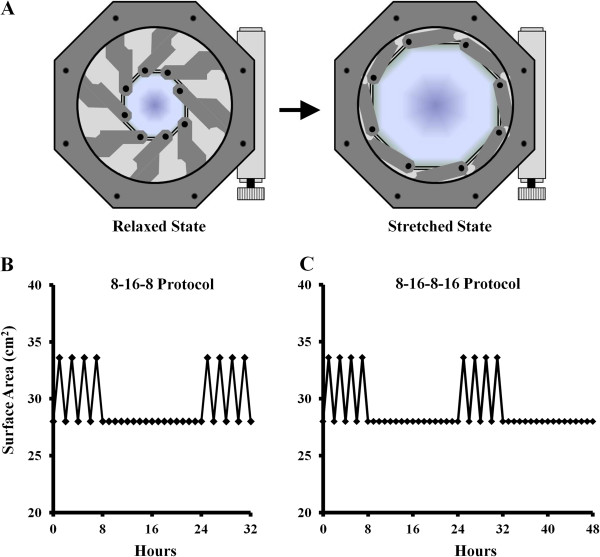
**Cell-stretching device and strain protocols. (A)** Schematic representation of mechanical stretching device used to apply low-frequency, high-magnitude strains. Graphical representation of the 8 hours/16 hours/8 hours stretch protocol **(B)** and the 8 hours/16 hours/8 hours/16 hours **(C)** stretch protocol applied to cells.

**Figure 3:**
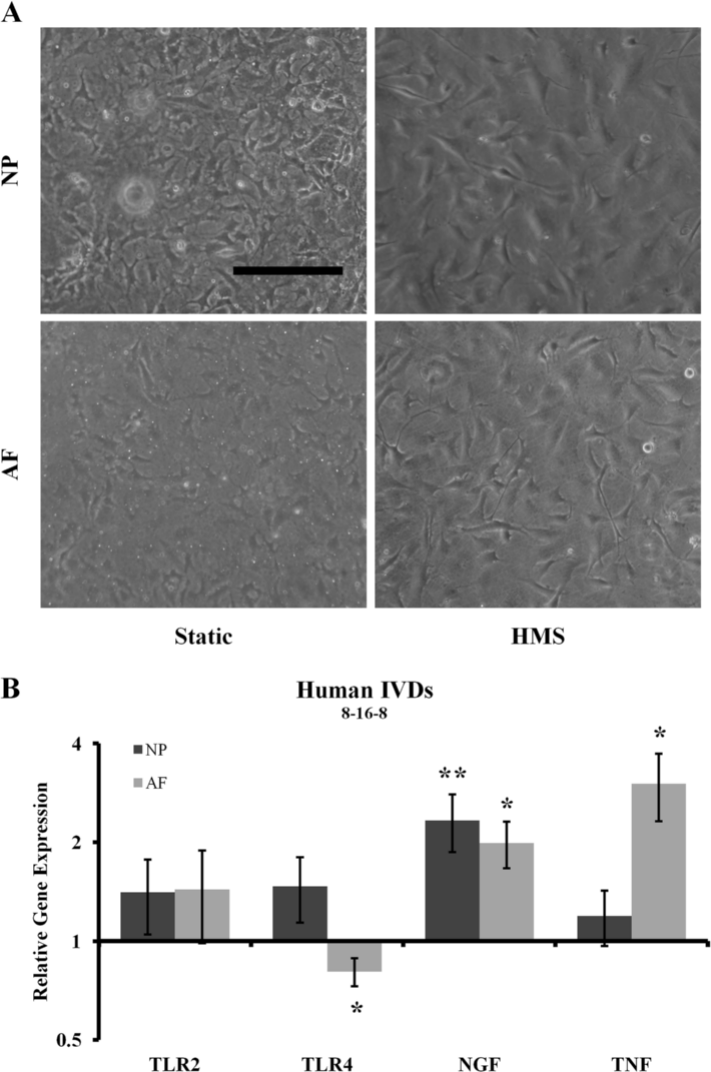
**Application of the 8 hours/16 hours/8 hours stretch protocol to human intervertebral disc cells. (A)** Representative morphological images of static and high mechanical strain (HMS) cultured human annulus fibrosus (AF) and nucleus pulposus (NP) cells. Scale bar: 200 μm. **(B)** Gene expression analysis of both AF and NP cells immediately after the stretch protocol ended. IVD, Intervertebral disc; NGF, Neuronal growth factor; TLR, Toll-like receptor; TNF, Tumour necrosis factor. Error bars represent SEM. Three independent experiments were performed. *P < 0.05 and **P < 0.01 by Student’s *t*-test.

**Figure 4:**
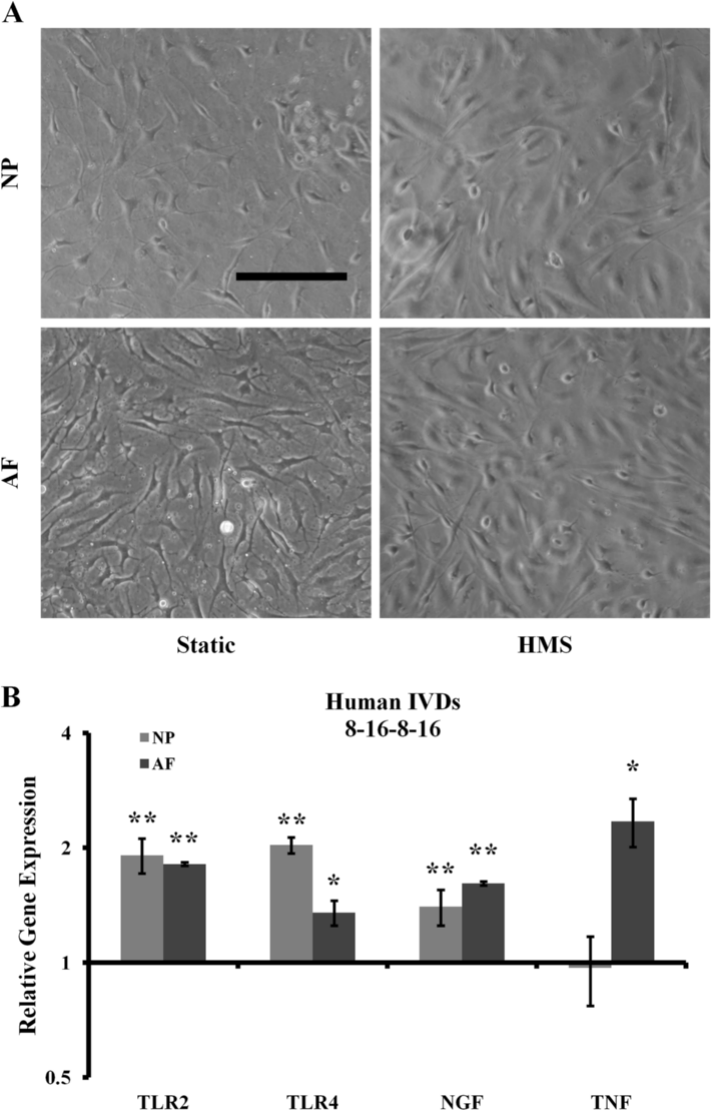
**Application of 8 hours/16 hours/8 hours/16 hours stretch protocol. (A)** Representative morphological images of human static and high mechanical strain (HMS) cultured annulus fibrosus (AF) and nucleus pulposus (NP) cells. Scale bar: 200 μm. **(B)** Gene expression analysis of both NP and AF cells after the additional 16-hour resting period. IVD, Intervertebral disc; NGF, Neuronal growth factor; TLR, Toll-like receptor; TNF, Tumour necrosis factor. Error bars represent SEM. Three independent experiments were performed. *P < 0.05 and **P < 0.01 by Student’s *t*-test.

To test whether HMS could initiate gene expression associated with IVD degeneration and discogenic pain, IVD cells were cultured in HESR dishes mounted on an iris-like stretching device (Figure 2A). Human AF and NP cells were subjected to two different stretch protocols, in each of which low-frequency cyclical strain (20%) was applied with different resting periods as outlined in Figures 2B and 2C. NP and AF cells subjected the 8 hours/16 hours/8 hours (Figure 3A) and 8 hours/16 hours/8 hours/16 hours (Figure 4A) stretch protocols did not display any striking differences in morphology compared to the static controls. Under the 8 hours/16 hours/8 hours stretch protocol, gene expression analysis revealed no significant changes in TLR2 in either the NP or AF cells (Figure 3B). TLR4 expression did not change in NP cells, there was minor, consistent downregulation in AF cells (1.25- ± 0.15-fold; *P* = 0.046). This stretch protocol promoted significant upregulation of NGF gene expression in both NP and AF cells (2.33- ± 0.93-fold and 1.99- ± 0.65-fold, respectively; *P* = 0.0099 and *P* = 0.032, respectively), as well as significant upregulation of TNF expression in AF cells (3.02- ± 1.41-fold; *P* = 0.027), compared to static controls (Figure 3B). Under the 8 hours/16 hours/8 hours/16 hours stretch protocol (Figure 4), gene expression analysis revealed significant upregulation of TLR2 (1.91- ± 0.20-fold and 1.81 ± 0.02-fold, respectively; *P* = 0.002 and *P* = 0.004, respectively), TLR4 (2.03- ± 0.10-fold and 1.35- ± 0.10-fold; *P* = 0.002 and *P* = 0.01, respectively) and NGF (1.40- ± 0.15-fold and 1.61- ± 0.02-fold; *P* = 0.008 and *P* = 0.0012, respectively) in both NP and AF cells, respectively, compared to static controls (Figure 4B). TNF expression was also significantly upregulated (2.35- ± 0.34-fold; *P* = 0.011), but only in AF cells.

### Conditioned media from high mechanical strain culture induces neurite outgrowth in PC12 cells

**Figure 5:**
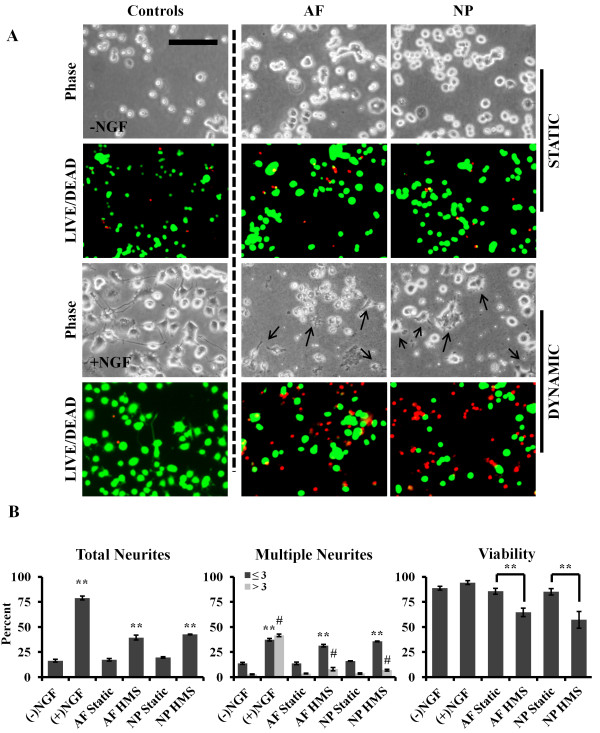
**Media from nucleus pulposus and annulus fibrosus cells cultured under high mechanical strain promote neurite outgrowth of PC12 cells. (A)** Conditioned media from static and high mechanical strain (HMS) nucleus pulposus (NP) and annulus fibrosus (AF) cells were incubated with PC12 cells, and neurite outgrowth and viability were observed and compared to vehicle (−NGF) and neural growth factor (NGF)-treated (+NGF) controls. Black arrows in phase images indicate neurites. Scale bar: 200 μm. **(B)** Quantification of total and multiple neurite outgrowth and viability. Error bars represent SEM three independent experiments were performed. *P < 0.05, **P < 0.01, and #*P* < 0.05 (all by Student’s *t*-test) for samples with three or more neurites per cell body. All samples were compared to –NGF controls.

To determine whether application of HMS to human IVD cells could stimulate secretion of factors that stimulate neuronal growth, collected conditioned media were applied to PC12 cells. The PC12 cell line was derived from rat adrenal pheochromocytoma and expresses receptors for, and responds to, βNGF [24], making this cell line useful for neuronal differentiation and axon growth studies [25,26]. Control PC12 cells without NGF remained small, circular and somewhat clustered, whereas those treated with 50 ng/ml of NGF became more flattened and spread out and grew multiple long neurite extensions (Figure 5A; phase image panels). PC12 cells appeared similar to controls when cultured in SS-conditioned media from both AF and NP cells. PC12 cells cultured in conditioned media from HMS-cultured AF and NP cells developed neurite extensions (Figure 5A; –NGF; black arrows).

PC12 cells cultured in HMS-conditioned media consistently looked grainy in appearance, with some debris in the dishes; therefore, we assessed their viability (Figure 5A). Quantification revealed significant cell death in PC12 cells exposed to HMS-conditioned media from both AF culture (64.6% ± 4.2% viable; *P* = 0.003) and NP culture (57.2% ± 8.4% viable; *P* = 0.0018) compared to vehicle-treated control (88.7% ± 1.87% viable) or NGF-treated control (94.3% ± 1.9% viable) (Figure 5; LIVE/DEAD image panels). No difference in viability was observed for cells cultured in SS-conditioned media from AF culture (85.6 ± 2.9% viable) and NP culture (85.2 ± 3.2% viable) compared to vehicle- and NGF-treated controls.

Quantification of neurite outgrowth revealed that 78.9% ± 1.8% of cells (*P* = 0.00039 vs. control) contained neurite extensions after treatment with NGF, whereas culture of cells with both HMS AF and NP media resulted in 39.4% ± 2.4% and 42.6% ± 3.8% of cells with neurites, respectively (*P* = 0.00078 and *P* = 0.0039 vs. control, respectively) (Figure 5B). These data were all significantly greater than vehicle-treated controls, in which 16.4% ± 1.26% of cells sprouted neurites. Cultures of PC12 cells with SS-conditioned media from both AF and NP cells resulted in neurite outgrowths (17.2% ± 3.81% and 19.6 ± 6.65%, respectively) similar to those in vehicle-treated controls. When we examined neurite density per cell, we found that NGF caused 37.3% ± 1.27% of PC12 cells to have three or fewer neurites per cell and 41.6% ± 1.19% to have more than three neurites per cell. Both of these percentages were significantly increased compared to vehicle controls (*P* = 0.0026 and *P* = 0.000015, respectively) (Figure 5B). Conditioned media from HMS-cultured AF and NP cells induced a significant increase in the proportion of cells containing three or fewer neurites per cell (31.4% ± 1.33% and 35.7% ± 3.35%; *P* = 0.00021 and *P* = 0.0012, respectively), as well as in the proportion containing more than three neurites per cell (7.96% ± 1.6% and 6.93% ± 0.93%; *P* = 0.002 and *P* = 0.00027, respectively) compared to vehicle-treated controls (Figure 5B). No differences were observed in neurite outgrowth for cells cultured with the static culture conditioned media from either AF or NP cells compared to vehicle controls.

### High mechanical strain with extended rest promotes cytokine secretion from human IVD cells

**Figure 6:**
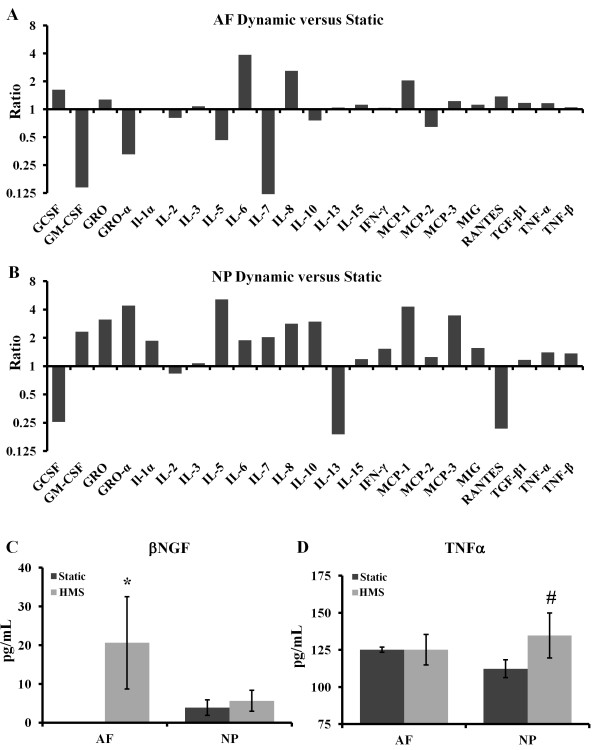
**Cytokine analysis of conditioned media.** Cytokine array blots were used to compare high mechanical strain (HMS) conditioned media of nucleus pulposus (NP) cells **(A)** and annulus fibrosus (AF) cells **(B)** with their respective static controls. Quantitation was carried out by performing enzyme-linked immunosorbent assays with conditioned media for neural growth factor (NGF) **(C)** and tumour necrosis factor α (TNFα) **(D)**. GCSF, Granulocyte colony-stimulating factor; GM-CSF, Granulocyte-macrophage colony-stimulating factor; GRO, growth-related oncogene; IFN, Interferon; MCP, monocyte chemoattractant protein; MIG, monokine induced by γ interferon; IL, Interleukin; TGF, Transforming growth factor. Error bars represent SEM. Three independent experiments were performed. *P < 0.05 and #*P* = 0.0504 by Student’s *t*-test (the latter statistic approaching significance).

Antibody arrays and enzyme-linked immunosorbent assays (ELISAs) were used to search for factors that could be responsible for the observed effect on PC12 cells. Conditioned media were incubated on cytokine antibody array blots to compare HMS-cultured AF and NP media to their respective static control media. HMS of AF cells caused decreases in the secretion of multiple cytokines, and many did not change compared to static cultured controls (Figure 6A). In contrast, HMS culture of NP cells caused increased secretion of the majority of factors probed (Figure 6B). In both NP and AF cells, HMS caused increased secretion of growth-related oncogene (GRO), IL-6, IL-8, IL-15, monocyte chemoattractant protein 1 (MCP-1), MCP-3, monokine induced by γ interferon (MIG), transforming growth factor β1 (TGFβ1) and TNFα. There was no overlap in any downregulated cytokines between the two groups.

In addition to the cytokine array, ELISAs were carried out to determine the accumulated NGF and TNFα concentrations in conditioned media. HMS of AF cells resulted in strong induction of NGF production, but no change in TNFα concentration. HMS AF media contained 20.61 ± 11.1 pg/ml NGF compared to levels below the detection limit in the static AF media (Figure 6C), which was statistically significant (*P* = 0.026). AF HMS-cultured media contained 125.1 ± 1.74 pg/ml TNFα compared to 125.3 ±10.3 pg/ml in the static AF media (*P* = 0.987) (Figure 6D). In contrast, stretching of NP resulted in an increase of TNFα, but not of NGF, in the medium. HMS NP cultures contained 5.64 ± 2.7 pg/ml NGF compared to 3.91 ± 2.01 pg/ml βNGF in the static NP media (Figure 6C). HMS media from NP cells contained 134.7 ± 15.2 pg/ml TNFα compared to 112.3 ± 6.03 pg/ml in the static NP media (*P* = 0.0504) (Figure 6D), which approached statistical significance.

## Discussion

Excessive mechanical loads can promote inflammatory and cytokine responses, factors implicated in spinal disc degeneration and low-back pain. To address the molecular and cellular effects of adverse mechanical strain, isolated human AF and NP disc cells were subjected to high-magnitude strains with a novel dynamic culture device coupled to a HESR culture dish. Gene expression, inflammatory and cytokine responses and conditioned media were analysed, which revealed upregulation of TLR2, TLR4, NGF and TNF gene expression, as well as higher levels of secreted GRO, IL-6, IL-8, IL-15, MCP-1, MCP-3, MIG, TGFβ1 and TNFα. Furthermore, conditioned media from HMS-cultured AF and NP cells were able to promote significant neurite outgrowth in PC12 cells. The conditioned media also caused a significant increase in cell death compared to static, NGF- and vehicle-treated controls. These data indicate that adverse mechanical strain can directly cause NP and AF secretion of factors associated with disc degeneration and discogenic pain and may have neurotoxic effects.

The primary purpose of IVDs is to mechanically bear and distribute load. Because of the heterogeneous nature of the NP, AF and surrounding cartilage endplates, discs experience complex loading. In loaded discs, NP cells generally experience hydrostatic and shear stresses, and AF cells are exposed to tensile strain [27,28]. These mechanical stresses change with aging and degeneration whereby the discs’ extracellular matrix integrity becomes compromised [29,30]. Changing mechanical forces can expose the AF and NP cells to adverse strains. Indeed, both NP and AF cells have been shown *in vitro* to respond to cyclic tensile loading. Mechanical compression of varying magnitudes can alter gene expression in NP cells [31], and mechanical stretch at higher frequencies (0.33 to 1.0 Hz) can differentially regulate anabolic and catabolic gene expression in AF cells [32,33]. The present study goes one step further in showing that significantly lower frequency (about four orders of magnitude lower) mechanical stimulation at high magnitude can both alter gene expression and promote cytokine, inflammatory and neurogenic signals from both AF and NP cells. These findings suggest that HMS, occurring during disc degeneration, could further accelerate degeneration and promote neuronal infiltration to the disc, which is thought to contribute to discogenic pain.

Several studies have found that TLR signalling is important in OA and cartilage degeneration and pain. Both TLR2 and TLR4 are highly expressed in varying levels of osteoarthritic chondrocytes [34,35], as well as in synovial tissue of rheumatoid arthritis patients [36]. Moreover, there is emerging evidence for a direct role in TLR2/TLR4 signalling to activate nuclear factor κB (NF-κB) and downstream cytokine cascades, which can result in chronic arthritis pain [37,38]. In addition, TLR signalling plays an important role in cytokine and chemokine secretion [10]. TLRs can promote production of IL-1, IL-6, IL-8, MIG, GRO and TNFα [39]. High mechanical stimulation of AF and NP cells not only induced upregulation of TLR2 and TLR4 gene expression but also induced secretion of the very same cytokines. All of these factors were found to be present in conditioned media from the adversely strained AF and NP cells, and PC12 cells exposed to these media formed neurites. MCP-1 and MCP-3 were both present in conditioned media from stretched AF and NP cells, and these factors have been associated with inflammatory neuropathic pain [40,41]. Taken together, the findings in the present study indicate that high-magnitude, low-frequency stimulation induces a coordinated TLR, cytokine and pronociceptive response by the IVD cells. Further experiments designed to dissect the relationship between TLR2/TLR4 signals and cytokine upregulation are required to better understand this correlation and its role in discogenic pain.

Inflammation and cytokine/chemokine release have an important direct role in spinal disc degeneration. Investigators in several recent studies have reported that IL-1, TNFα and chemokine (C-C motif) ligand 5/regulated on activation, normal T cell expressed and secreted (RANTES) were found to be expressed in degenerated discs [6,42,43]. Expression of these factors leads to a catabolic shift within disc tissue whereby AF and NP protease activity becomes increased and may lead to degeneration [44,45]. In the present study, we found upregulation of not only cytokines but also NGF gene and protein expression in AF and NP cells exposed to HMS. We also observed distinct correlations between cytokines, NGF, NGF receptors and TLR signalling [46-48]. The presence of these factors in HMS-conditioned media, combined with effects on PC12 differentiation, suggests that this type of mechanical strain at the cellular level can play a potentially strong role in disc degeneration, disc innervation and discogenic pain.

When conditioned media from HMS and SS cultures were applied to PC12 cells, a grainy appearance and particle debris were consistently observed in culture dishes with HMS media. Although a significant number of cells developed neurites, viability assays confirmed significant cell death. High levels of cytokines present in the HMS-conditioned media likely mediated the observed PC12 cell death. TNFα [49] and IL-1 [50] have been shown to induce cell death in PC12 cells, and both were present in the HMS media. TNFα and IL-1β are also known to have neurotoxic properties whereby they work in concert with other cytokines to elicit their apoptotic effects [51]. PC12 cells, however, are derived from a rat cell line, and HMS-conditioned media in this study arose from primary human cells. It is conceivable that PC12 cells are more sensitive to the factors released by human cells. Certainly, cell death factors must be analysed in the conditioned media of HMS-cultured cells. Future studies will determine which factors in the HMS media are responsible for the neurotoxicity and through which mechanisms they act.

Because of its highly expandable culture surface, the dynamic culture device was initially used as a means to culture large populations of primary chondrocytes [15] without the need for passaging, enabling phenotype retention. The device is also capable of applying low-frequency dynamic strain to cultured cells, however, which has been shown to modulate differentiation of mesenchymal stem cells [19] and myoblastic cells [18]. Several reports have suggested that cell death can be induced by applying high cyclic stretch magnitudes over 20% combined with frequencies above 0.5 Hz. Indeed, cyclic strain greater than 20% with frequencies in excess of 0.5 Hz can induce apoptosis in AF cells of the disc, human endothelial cells and tendon cells [52-54]. An advantage of using this culture device is that high-magnitude strain can be achieved without inducing cell death due to the very low frequencies applied. Another advantage of using our culture device is that functionalization of the silicone culture surface allows for coating with molecules which can support the growth and phenotype of the cell being cultured. For example, our culture surfaces are typically coated with collagen type I for ease and cost-effectiveness. However, this type of culture surface can be modified and coated with matrix extracts from which the isolated cells originate, thus providing a biomimetic microenvironment coupled with mechanical stimulation [16]. One other limitation of this study is that cells are strained in monolayer culture. Although this allows for direct analysis of mechanical strain solely on the cultured cells, it does not reflect overload strains on disc cells in their native three-dimensional environment. Whether the cells undergoing HMS are subject to the same increased strains as those of disc cells within degenerate IVDs remains to be determined. Future experiments in which isolated cells are cultured on IVD matrix extracts or within three-dimensional scaffolds [55] or in which whole human discs are cultured within a bioreactor [56,57] exposed to varying mechanical overload stimulation levels may provide further insights into the cellular and whole-tissue effects of high magnitude strain.

## Conclusions

HMS culture of human AF and NP cells *in vitro* drives upregulation of the cytokine, inflammatory and neurotrophic molecules TLR2, TLR4, NGF, TNF, GRO, IL-6, IL-8, IL-15, MCP-1, MCP-3, MIG, TGFβ-1 and TNFα. These factors are associated with DDD and low-back pain. This study provides striking evidence for a direct link between high tensile cellular strain, secretory factors, neoinnervation and potential degeneration and discogenic pain *in vivo*.

## Abbreviations

AF: Annulus fibrosus; DDD: Degenerative disc disease; DMEM: Dulbecco’s modified Eagle’s medium; GRO: Growth-related oncogene; HMS: High mechanical strain; IL: Interleukin; IVD: Intervertebral disc; MCP: Monocyte chemoattractant protein; NGF: Neuronal growth factor; NP: Nucleus pulposus; OA: Osteoarthritis; PC12: Pheochromocytoma cell line 12; TGF: Transforming growth factor; TLR: Toll-like receptor; TNF: Tumour necrosis factor.

## Competing interests

One of the authors (TMQ) is a shareholder in the Cytomec GmbH, which makes the iris-like devices for cell culture on the high-extension surfaces used in this study. There are no further conflicts of interest to declare.

## Authors’ contributions

RG, DHR and EK performed the experiments. DHR, RG, TMQ, JO, LSS and LH designed the research study. RG, DHR and EK analysed the data. RG, DHR and EK prepared all figures. RG, DHR and LH wrote the first draft of the manuscript. All authors participated in the interpretation of the study results. All authors were involved in drafting and revising the manuscript. All authors approved the final version of the manuscript for submission.
